# Silver Nanowire Ink for Flexible Circuit on Textiles

**DOI:** 10.3390/mi10010042

**Published:** 2019-01-09

**Authors:** Dexi Du, Xing Yang, Yonglan Yang, Yuzhen Zhao, Yuehui Wang

**Affiliations:** 1 School of Materials and Energy, University of Electronic Science and Technology of China, Chengdu 610054, China; dudexi_work@foxmail.com (D.D.); shirleywyh@126.com (X.Y.); 2 Department of Materials and Food, University of Electronic Science and Technology of China Zhongshan Institute, Zhongshan 528402, China; wyhzsedu@126.com; 3 Department of Materials Science and Engineering, Tsinghua University, Beijing 100084, China; zhaoyz@mail.tsinghua.edu.cn

**Keywords:** silver nanowires, textile, conductive coating, conductivity

## Abstract

Low cost electronics implemented in textiles could pave the way to a fully new generation of smart products in the fields of healthcare, sport, fashion, and safety. Although many methods have found their way into the market, many problems still need to be solved and much progress has to be made to enable the commercial exploitation of such products. In this paper, silver nanowires of 60–100 nm in diameter and 8–15 μm in length were achieved by the polyol solvothermal method, and aqueous silver nanowire conductive inks were prepared with the synthesized silver nanowires as the conductive phase, in the presence of polyaniline, guar, and hydrochloric acid. The conductive inks were printed on cotton fabric substrate by screen printing process. The effects of the amount of silver nanowires, layers of coating, and treatment temperature on the microstructure and electrical properties of samples were investigated by scanning electron microscopy and the four-point probe method. The results show that the conductivity and densification of the samples increased with increased amount of silver nanowires, layers of coating, and treatment temperature. The heat treatment helped to improve densification of the silver nanowires and conductivity of the sample. The resistance of the samples increased after bending due to loosening of the overlap between the silver nanowires.

## 1. Introduction

From the synthesis to the application of nano-silver, researchers have done a lot of work on nano-silver. The morphologies of nano-silver are varied, including nanospheres, nanorods, nanorice, nanobipyramids, nanosheets, nanowires, and so on [1,2,3,4,5]. Prompted by current research, the applications of nano-silver in the fields of conductive ink, catalysts, and bactericide have attracted the attention of many researchers [6,7,8,9]. Due to its low resistivity, low sintering temperature, toughness, and low price, nano-silver has been applied to conductive ink by a large number of researchers. Most nano-silver inks have been prepared with nanospheres or silver complexes. However, the resistance of conductive inks configured with nanospheres or silver complexes is high. The reason is the high contact resistance among the nanostructures [5,6,7,8,9]. Silver nanowire, with its one-dimensional structure, has good electrical and thermal conductivity, flexibility, etc. The preparation and application of silver nanowires have attracted the interest of researchers [10,11,12,13,14,15].

Generally, conductive inks configured with nanospheres are used for flexible circuits or electrodes that do not require transparency [10]. In contrast, nanowire ink is used to make flexible, conductive, transparent thin films or electrodes, especially for solar cells, displays, etc. [11,12,13]. The sheet resistance and light transmittance can be comparable to indium tin oxides (ITO) [14], and these nanowire products have excellent flexibility and low temperature treatment characteristics that ITO does not have. In particular, the concept of wearable electronic consumer products is becoming more and more popular. To realize large-scale marketization of wearable electronic consumer products, it is necessary to improve the manufacturing technology of flexible circuits. Conductive inks are the key to achieving flexible circuits. The conductive ink is coated onto flexible substrates, such as fabric, paper, polyethylene terephthalate (PET), and polyimide (PI), by silkscreen printing, inkjet printing, spraying, vacuum extraction, and the filtration transfer method to make flexible, conductive pathways or transparent conductive films, which are then applied to wearable electronic products [15,16,17,18,19]. The main factors affecting the electrical properties of conductive inks are the shape and size of the nanoparticles, the solid content, the printing process, the sintering process, and the pretreatment of the substrate surface [15,16,17,18,19,20,21]. In our group, we prepared conductive inks with silver nanowires as the conductive phase and investigated the effects of the mass fraction of silver nanoparticles and the sintering process on the microstructure and electrical properties of samples [22].

Until now, not much study of silver nanowires as fillers of inks applied on textile substrates has been reported, and the existing studies have mainly been done in the field of flexible transparent conductive films. In this paper, silver nanowires (AgNWs) were achieved by the polyol solvothermal method, and aqueous silver nanowire conductive inks in the presence of polyaniline, guar, and hydrochloric acid were prepared and printed on cotton fabric substrate. The effects of the amount of silver nanowires, number of coating layers, and heat treatment temperature on the microstructure and electrical properties of samples were investigated.

## 2. Materials and Methods

### 2.1. Materials

Silver nitrate (≥99.8%) was purchased from Guangzhou Jinhuada Chemical Reagent Co., Ltd. (Guangzhou, China); poly(vinylpyrrolidone) (PVP, K30, Mw ≈ 10,000) was purchased from Jinan Jiage Biological Technology Co., Ltd. (Jinan, China); ferric chloride hexahydrate (AR) was purchased from Shanghai Hongshun Biological Technology Co., Ltd. (Shinghai, China); and ethylene glycol (AR) and ethanol absolute (AR) were purchased from Jinan Liyang Chemical Co., Ltd. (Jinan, China). Aniline (AR) and hydrochloric acid (HCl, AR) were purchased from Tianjin Yongda Chemical Co., Ltd. (Tianjin, China), and ammonium persulfate (AR) and nitric acid (HNO_3_, AR) were purchased from Tianjin Baishi Chemical Co., Ltd. (Tianjin, China). Guar gum (AR) was purchased from Henan Onist Food Co., Ltd. (Zhengzhou, China). All chemicals were used as received.

### 2.2. Method

AgNWs were prepared by polyol solvothermal method [16,23]. Both HNO_3_ (8.7 mL) and aniline (5.8 mL) were dissolved in deionized water (200 mL), and ammonium persulfate (14.26 g) was dissolved in deionized water (50 mL). The above two solutions were mixed while stirring and reacted at room temperature for 4 h. The reaction solution gradually changed from transparent solution to dark green viscous liquid, thus polyaniline solution was obtained. Guar gum 2 wt.% was slowly added into 100 mL deionized water while stirring at room temperature for 4 h to obtain guar gum colloid, which is a pale yellow, viscous liquid.

The typical preparation process of the silver nanowire ink is as follows: 30 mL of 2.2 wt.% nanowires, 6 mL of polyaniline, 8 mL of guar gum, and 3 mL of HCl solution (30 mmol·L^−1^) were mixed and magnetically stirred for 20 min. AgNW conductive inks were coated onto the fabric cloth (3 × 3 cm^2^) by screen printing process (printing by manual, 200/inch nylon wire mesh screens, scraper angle: 75°) and placed on a hot plate to heat treat, then cooled to room temperature. To prepare multi-layer AgNW coatings, the samples were left at room temperature for 20 min after each coating, then coated with the next layer and treated at room temperature. The microstructures of all of samples were examined by Zeiss MERLIN VP Compact (JSM-6460) scanning electron microscope (SEM, Carl Zeiss AG, Jena, Germany). The sheet resistance of the sample was measured using 4-point probe instrument ST2253 (Suzhou Jingge Electronics Co., Ltd., Suzhou, China). The sheet resistance of each sample was measured at 8 points in different places, and the mean value was calculated. measurement. X-ray diffraction (XRD, DIFFRRACTOMETER, Rigaku Co., Japan) was used to measure the phase structures.

## 3. Results and Discussions

**Figure 1:**
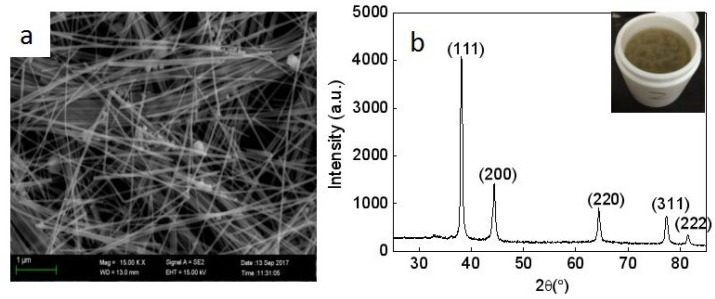
(**a**) Scanning electron microscope (SEM) and (**b**) XRD analysis of silver nanowires. The inset photo is as-synthesized AgNW solution.

An SEM image (Figure 1a) and the XRD spectrum (Figure 1b) of the purified silver nanowires are shown in Figure 1. The photo of as-synthesized silver nanowires is inset in Figure 1b. Silver nanowires of 60–100 nm in diameter and 8–15 μm in length were achieved (Figure 1b). As can be seen from the XRD spectrum shown in Figure 1b, the XRD peaks are at (111), (200), (220), (311), and (222), from which it can be identified that the nanowires had a face-centered cubic (fcc) crystal structure. The color of as-synthesized silver nanowire solution was gray.

**Figure 2:**
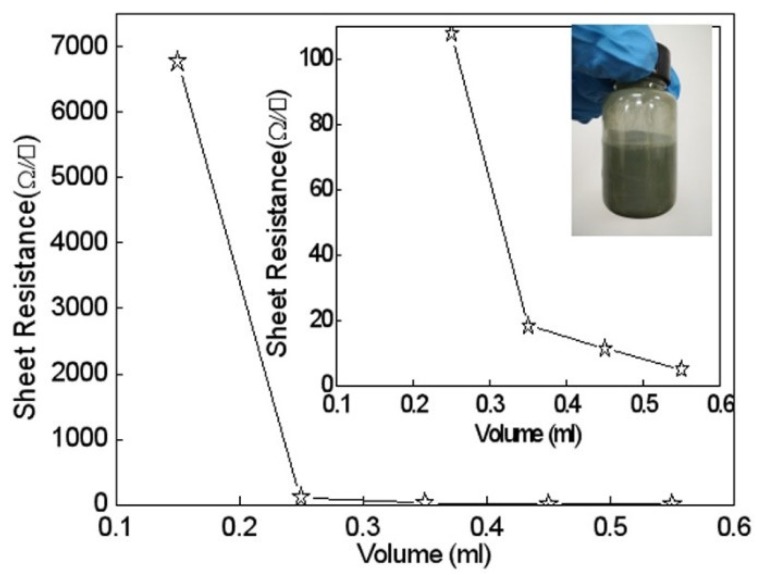
Relationship between sheet resistance of AgNW coating and amount of AgNWs. The inset is a local enlarged image and photo of AgNW ink.

Figure 2 shows the relationship between the sheet resistance of the silver nanowires coatings and the amount of AgNW ink. The amounts of AgNW ink shown in Figure 2 are 0.15, 0.25, 0.35, 0.45, and 0.55 mL, which were used to print a 7 mm × 100 mm conductive path on the fabric cloth. The treatment temperature of the samples was 35 °C. The inset is the local enlarged image and the photo of AgNW ink. As seen in Figure 2, when the amount of AgNW ink was 0.15 mL, the sheet resistance of AgNWs coating was 6700 Ω·sq^−1^. When the amount of AgNWs ink was 0.25 mL, the sheet resistance dropped sharply to 107 Ω·sq^−1^, indicating that the electrical conductivity of AgNW ink was in accordance with percolation theory. When the amount of AgNWs ink increased to 0.35 mL, the sheet resistance of AgNWs coating decreased to 18.43 Ω·sq^−1^. When the amount of AgNWs ink was 0.45 mL, the sheet resistance of AgNWs coating was 11.45 Ω·sq^−1^. When the amount of AgNWs ink increased to 0.55 mL, the sheet resistance of silver nanowires coating was 5.10 Ω·sq^−1^.

It is clear that the conductive behavior of AgNW ink conforms to the percolation model. After exceeding a certain loading level of conductive ink, the electrical property improves marginally. The decrease in electrical surface resistivity is due to the increased amount of AgNWs. The percolation model illustrates the relationship between the quantity by weight of added conductive materials and the achieved electrical resistivity. The percolation behavior depends on the aspect ratio of the conductive materials, the material size, and its density and chemical structure [17]. The electrical conductivity of the film can be improved by application of the proper content of AgNWs, with high contents having little influence on the electrical conductivity, because effective conductive networks have already been formed in the film.

**Figure 3:**
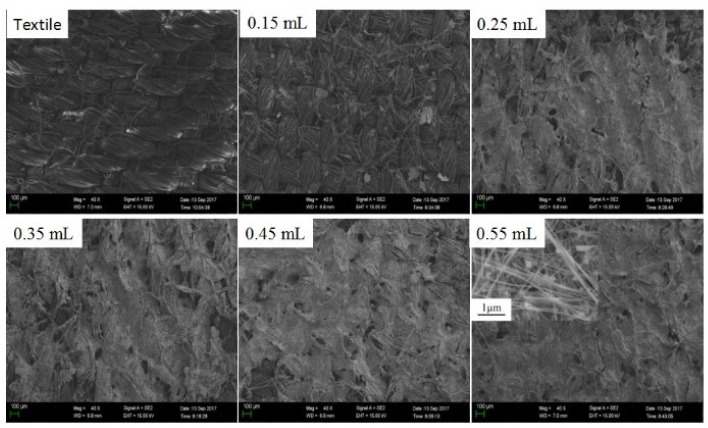
SEM images of AgNW coatings with different amounts of AgNW inks.

Figure 3 shows SEM images of the samples. The inset is an SEM image of the pure AgNWs. As seen in Figure 3, only a small amount of AgNWs cover the surface of the fiber substrate when the amount of AgNWs is 0.15 mL; the conductive coating is loose and the conductive path is not good enough, so the sheet resistance is very high. With increasing of amounts of AgNWs, more AgNWs deposited on the surface of the textile, and more conductive paths were formed. The conducting contacts among the AgNWs were believed to be enhanced with increasing amount of AgNWs. As seen in Figure 3, some AgNWs coated the surface of the fibers to form a thin sheet.

**Figure 4:**
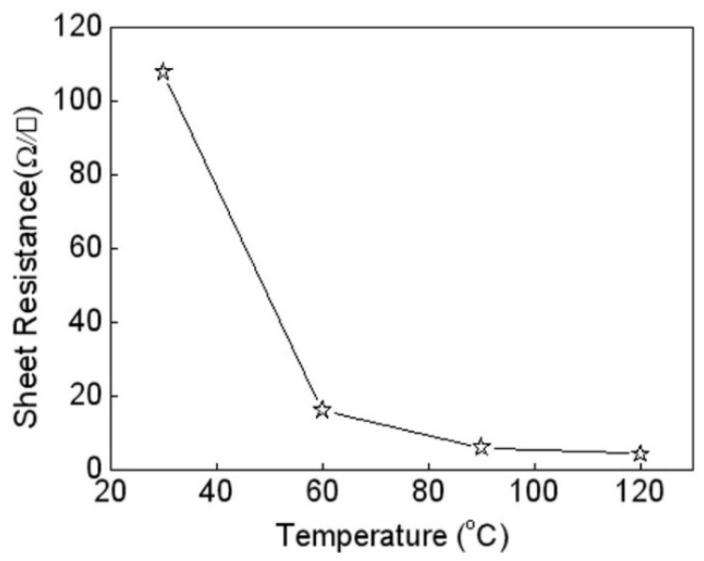
Relationship between the sheet resistance of AgNW coatings and treatment temperature.

Heat treatment is an important step in improving the conductivity of AgNW coatings. By the heat treatment process, the AgNW coating achieves a transition from low- or even non-conductivity to high conductivity. Figure 4 shows the relationship between the sheet resistance of AgNW coatings (0.25 mL AgNWs ink) and their treated temperature. It is clear that the sheet resistance dropped when the treatment temperature was raised from room temperature to 60 °C. After that, the sheet resistance of the coating slightly decreased with increasing treatment temperature, and the sheet resistance of the coating reached 4.31 Ω·sq^−1^ at 120 °C. These results indicate that there are some differences in the states of the percolation networks consisting of conductive materials which depend on the treatment temperature. The contact resistance at the interfaces between silver nanowires is considered to be strongly influenced by a compressive contact stress due to the thermal effect. In addition, HCl as a sintering accelerator was added into the silver nanowire ink, and HCl concentration slowly increased as the solvent evaporated. Due to the strong nucleophilicity of chloride ions, chloride ions gradually replaced the sodium citrate on the surface of the silver nanowires to adsorb to the surface of the nanowires [18]. As the drying process progressed, the nanowires gradually gathered together and contacted each other to form a continuous conductive path, so conductivity was enhanced.

**Figure 5:**
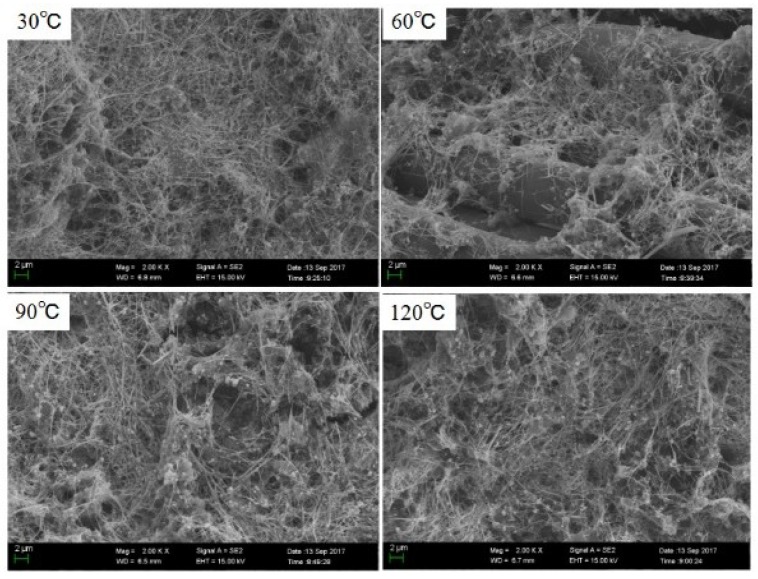
SEM images of AgNW coatings on the surface of fabric at different treatment temperatures.

Figure 5 shows SEM images of AgNW coatings on the surface of fabric at different treatment temperatures. In Figure 5, although the obvious differences in the distribution and morphology of AgNWs at different heat treatment temperatures cannot be observed, lots of AgNWs coated on the surface of the fabric were observed.

**Figure 6:**
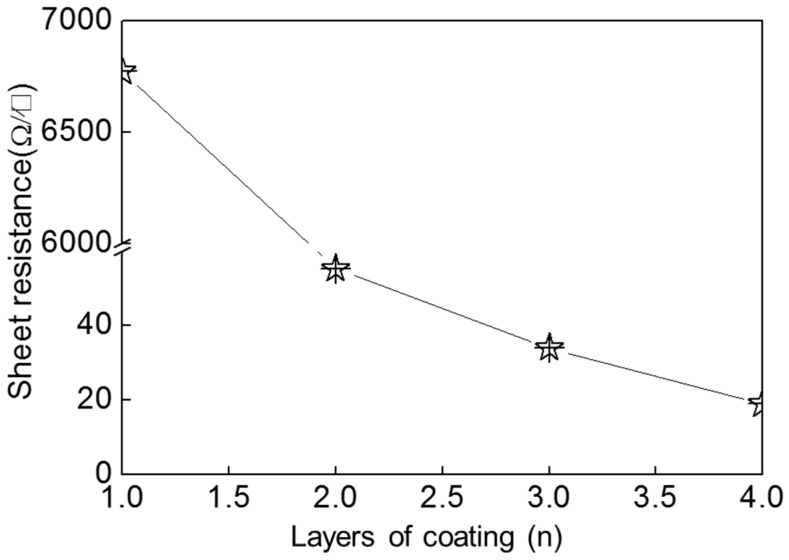
Relationship between sheet resistance of AgNW coatings and number of coating layers.

We also studied the effect of layer of coating on conductivity of AgNW coatings. We dropped 0.15 mL AgNW ink on the surface of fabric and repeated four times, and samples were naturally dried at room temperature. Figure 6 shows the relationship between the sheet resistance of AgNW coatings and the layers of coating. It is clear that the sheet resistance of the coating decreased significantly with increased layers of coatings. The sheet resistances of the coatings with two layers, three layers, and four layers were 55.41 Ω·sq^−1^, 34.09 Ω·sq^−1^, and 18.95 Ω·sq^−1^, respectively. It is clear that the conductivity of four-layer AgNW coatings with 0.15 mL is similar to that of a single AgNW coating with 0.35 mL. With increased number of coatings, more silver nanowires were deposited on the surface of the fabric and more conductive paths were formed, which improved the conductivity of the coating.

**Figure 7:**
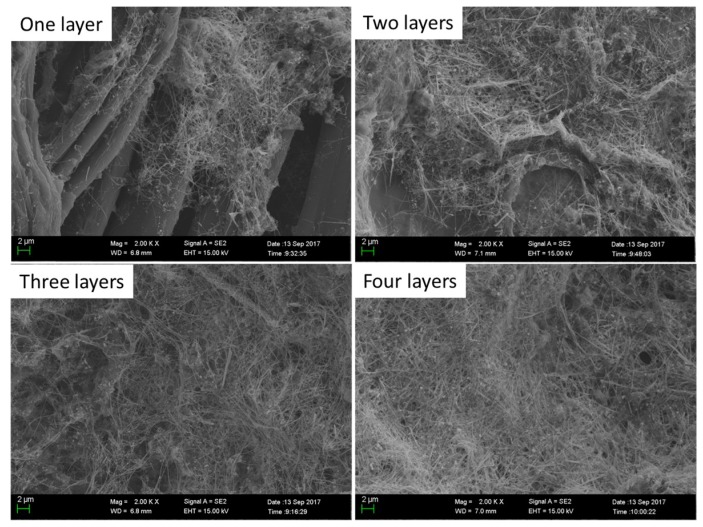
SEM images of silver nanowire coatings on the surface of fabric with different numbers of layers.

Figure 7 shows SEM images of AgNW coatings on the surface of fabric with different numbers of layers. As shown in Figure 7, a small number of AgNWs cover the surface of the fiber in the case of one layer of the AgNW coating. With increasing layers of AgNW coating, more and more AgNWs are deposited on the surface of the fiber.

**Figure 8:**
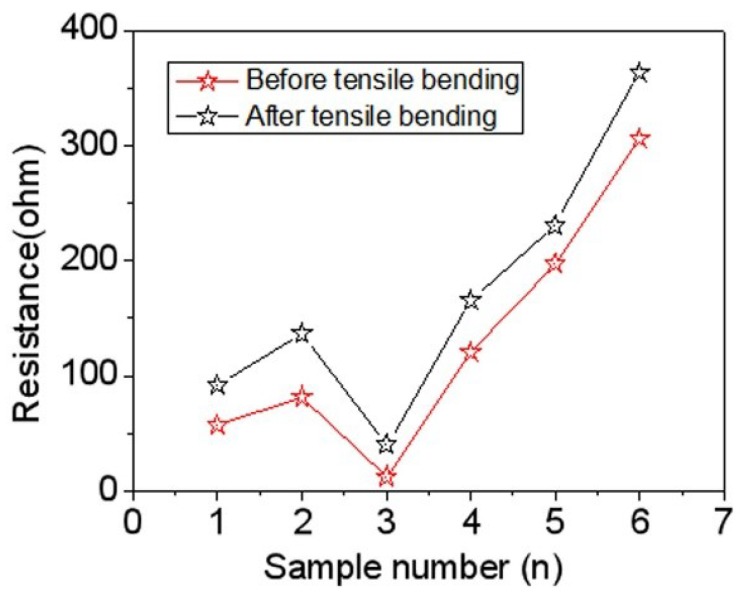
Relationship between resistances of silver nanowire coatings and bending times.

In order to study the change of resistance of the conductive coatings after bending, the resistance of the conductive coatings were measured, as shown in Figure 8. The change in the resistance of the conductive coating was observed after bending. The maximum rate of change was 36.3% and the minimum was 4.06%. The resistance of the samples increased after bending due to the loosening of the overlaps among the AgNWs.

**Figure 9:**
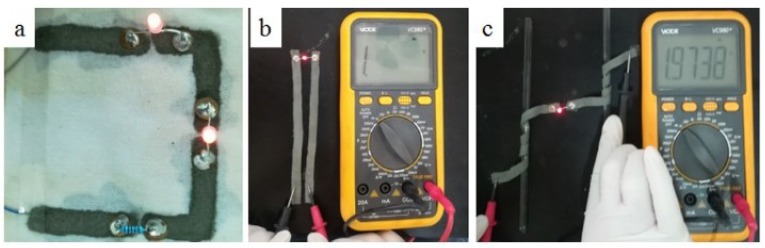
Three samples of the conductive loop with conductive ink, illuminating small LED lamp beads.

In order to verify the application of the AgNW inks, we printed a circuit diagram on a clean woven fabric, using the diode position of the multimeter as the power supply, and realized the construction of a two 0.06 W LEDs and 1 KΩ resistor circuit, as shown in Figure 9. It is clear that the LEDs lit up successfully (Figure 9a), meanwhile, the fabric still worked well when bent (Figure 9b,c).

## 4. Conclusions

We successfully synthesized AgNWs, and aqueous silver nanowire conductive inks were prepared using the as-synthesized silver nanowires as the conductive phase in the presence of polyaniline, guar, and hydrochloric acid. When the amount of silver nanowire ink was 0.55 mL, the sheet resistance of silver nanowire coating treated at 35 °C was 5.10 Ω·sq^−1^. With the increase of the heat treatment temperature, the sheet resistance reduced due to the effects of the heat. The sheet resistance of the coating heat treated at 120 °C reached 4.31 Ω·sq^−1^. With increasing layers of coating, the sheet resistance decreased. The sheet resistance of the coatings with two layers, three layers, and four layers were 55.41 Ω·sq^−1^, 34.09 Ω·sq^−1^, and 18.95 Ω·sq^−1^, respectively. The conductivity of four-layer AgNW coatings with 0.15 mL was similar to that of a single AgNW coating with 0.35 mL. The change in the resistance of the conductive cloth bands after bending was also measured: the maximum rate of change was 36.3% and the minimum was 4.06%. Meanwhile, the small LED lamp beads were successfully illuminated with the conductive cloth band. Therefore, our work provides some ideas for further research into wearable electronic devices.
